# Nilotinib in patients with systemic mastocytosis: analysis of the phase 2, open-label, single-arm nilotinib registration study

**DOI:** 10.1007/s00432-015-1988-0

**Published:** 2015-05-23

**Authors:** Andreas Hochhaus, Michele Baccarani, Francis J. Giles, Philipp D. le Coutre, Martin C. Müller, Andreas Reiter, Helene Santanastasio, Mimi Leung, Steven Novick, Hagop M. Kantarjian

**Affiliations:** 1 Abteilung für Hämatologie und Internistische Onkologie, Universitätsklinikum Jena, Erlanger Allee 101, 07740 Jena, Germany; 2 University of Bologna, Bologna, Italy; 3 Northwestern Medicine Developmental Therapeutics Institute, Chicago, IL USA; 4 Charité - University of Medicine Berlin, Berlin, Germany; 5 Medizinische Universitätsklinik, Medizinische Fakultät Mannheim der Universität Heidelberg, Mannheim, Germany; 6 Novartis Pharmaceuticals Corporation, East Hanover, NJ USA; 7 The University of Texas, MD Anderson Cancer Center, Houston, TX USA

**Keywords:** Nilotinib, Tyrosine kinase inhibitor, Systemic mastocytosis, KIT D816V, Aggressive systemic mastocytosis, Indolent systemic mastocytosis

## Abstract

**Purpose:**

Activating *KIT* mutations are part of the pathogenesis of systemic mastocytosis (SM). Nilotinib is a tyrosine kinase inhibitor that potently inhibits activated forms of KIT. This phase 2, open-label, single-arm study (CAMN107A2101; www.clinicaltrials.gov NCT00109707) evaluated nilotinib in patients with SM.

**Methods:**

Patients with SM [aggressive SM (ASM), indolent SM, or other] received nilotinib 400 mg twice daily. C-findings were collected retrospectively to assess response using criteria proposed after trial initiation. Response was evaluated using improvements in laboratory findings (for all patients) and ASM response criteria (for the ASM subgroup).

**Results:**

In 61 patients enrolled, the median nilotinib exposure was 232 days (range 3–1274 days) with a median follow-up of 34.7 months. In patients with ASM (*n* = 37), the overall response rate was 21.6 %. In the eight responders, all of whom had a *KIT* D816V mutation at any time, mast cell infiltration and tryptase level decreased by 70 % and 29.8 %, respectively; absolute neutrophil count increased by 94.7 %. Laboratory parameters also improved in the non-ASM subgroups. Overall survival at 24 months was 81.2 % (95 % CI 70.6–91.8 %) with median survival not yet reached. New or worsening grade 3/4 hematologic adverse events (AEs) included thrombocytopenia (10.3 %), anemia (10.0 %), and neutropenia (6.9 %). The most common grade 3/4 nonhematologic drug-related AEs were diarrhea (6.6 %) and headache (4.9 %). Eleven patients (9 with ASM, 2 with MCL) died, 10 due to progressive disease; 7 deaths occurred ≥28 days after treatment discontinuation.

**Conclusions:**

Nilotinib 400 mg twice daily was effective in some patients with SM, including patients with mutated *KIT* D816V.

## Introduction

Systemic mastocytosis (SM) is a myeloproliferative neoplasm characterized by the proliferation and activation of mast cells in various organs, including the skin, liver, spleen, and hematopoietic tissues, eventually resulting in impaired organ function (Valent et al. 2003). The clinical course in SM is heterogeneous and ranges from asymptomatic with a normal life expectancy to highly aggressive with decreased life expectancy (Quintas-Cardama et al. 2011). SM is rare; the annual incidence of new cases is 0.3 per 100,000 (Woodward 2003). The median overall survival is approximately 63 months overall, 198 months for patients with indolent SM (ISM), 24 months for patients with aggressive SM (ASM), and 2 months for patients with mast cell leukemia (MCL) (Lim et al. 2009b; Fuller 2012).

SM is classified based on the abnormal morphology, immunophenotype, and molecular characteristics of mast cells. The diagnostic algorithm for SM includes evaluating bone marrow by immunostaining for the presence of tryptase and/or KIT and immunophenotyping mast cells for expression of CD25 and/or CD2. Serum tryptase level and the presence of the *KIT* D816V mutation in blood or bone marrow are also evaluated. SM is then categorized as ISM (2 or more B-findings), ASM (1 or more C-findings), or MCL (at least 20 % mast cells on bone marrow aspirate smear) (Pardanani 2015; Horny et al. 2008).

There is no accepted standard therapy for patients with SM. Patients generally receive treatments intended to manage symptoms and improve quality of life, such as antihistamines for the relief of pruritus and flushing, proton pump inhibitors to treat gastrointestinal symptoms, or corticosteroids and/or analgesics for mitigating bone pain and other symptoms (Andersen et al. 2012; Valent et al. 2010; Pardanani 2015). Patients with advanced SM may receive treatment with interferon (IFN)-α or cladribine; these treatments also decrease symptoms but may not substantially reduce mast cell burden (Verstovsek 2013). Tyrosine kinase inhibitors (TKIs), such as imatinib, have demonstrated modest effects in SM, although primary resistance is common in patients with the *KIT* D816V mutation (Lim et al. 2009a; Pardanani 2012; Valent et al. 2010).

As many as 93 % of SM cases may harbor an activating D816V mutation in the catalytic domain of KIT (Garcia-Montero et al. 2006; Quintas-Cardama et al. 2011; Akin and Metcalfe 2004), a receptor tyrosine kinase expressed on the surface of mature mast cells and mast cell precursors (Lammie et al. 1994; Valent et al. 2003). The *KIT* D816V mutation induces downstream signaling that is independent of the KIT ligand stem cell factor (Furitsu et al. 1993) and is mediated through the signal transducer and activator of transcription 5 (STAT5) and phosphoinositol-3-kinase (PI3K) pathways (Harir et al. 2008). Constitutive KIT activation results in increased mast cell accumulation in the bone marrow and more aggressive disease (Valent et al. 2003; Lim et al. 2009b; Verstovsek 2013). The presence of the *KIT* D816V mutation is one of four minor criteria for the diagnosis of SM (Valent et al. 2003), and *KIT* D816V allele burden can be used to monitor residual disease in patients with SM (Erben et al. 2014). Additional *KIT* mutations as well as KIT-independent pathways (e.g., Lyn and Btk) have recently been implicated in the pathogenesis of SM (Gleixner et al. 2011; Schwaab et al. 2013; Orfao et al. 2007).

The TKI nilotinib was rationally designed to inhibit mutant forms of the BCR-ABL protein that display resistance to the TKI imatinib in patients with chronic myeloid leukemia (CML); both imatinib and nilotinib are approved for the treatment of CML. Nilotinib is also active against the KIT kinase in vitro (Weisberg et al. 2005; Manley et al. 2010). Recent data from a multicenter, phase 2, open-label registration trial demonstrated that nilotinib 400 mg twice daily continued to be safe and effective in patients with CML in chronic phase (Giles et al. 2013), accelerated phase (le Coutre et al. 2012), and blast crisis (Giles et al. 2012) who were resistant to or intolerant of prior therapies. Based on promising results from the first data analysis (Hochhaus et al. 2006), we evaluated the efficacy and safety of nilotinib 400 mg twice daily in patients with SM (with or without the D816V mutation) enrolled in the phase 2 nilotinib registration trial (CAMN107A2101, registered at www.clinicaltrials.gov as NCT00109707).

## Materials and methods

### Patient population

Enrollment criteria for the phase 2, multicenter 2101 trial have been previously described (H. M. Kantarjian et al. 2007). Briefly, adult patients with hematologic malignancies were recruited into 6 parallel treatment arms. Patients who met the standard disease criteria for SM (at least 1 major and 1 minor or 3 minor criteria for SM) (Valent et al. 2003) and needed treatment were recruited into the SM arm of the study and were assessed for efficacy according to a Simon two-stage minimax design (Simon 1989). The major criterion for SM is the presence of multifocal clusters of mast cells in the bone marrow. The minor criteria are the presence of spindle-shaped mast cells in the marrow, elevated serum tryptase levels, abnormal expression of CD2 or CD25, and the presence of the *KIT* D816V mutation (Valent et al. 2001). Key inclusion criteria included World Health Organization (WHO) performance status ≤2. Exclusion criteria included disease infiltration into the central nervous system and impaired cardiac function (including a left ventricular ejection fraction <45 %, congenital long QT syndrome, or QTc >450 ms).

Recruitment occurred between June 9, 2005, and March 31, 2006. Data on the significance of improvements in clinical findings (C-findings) as a measure of treatment response and clear definitions of the various types of SM were published after initiation of the study (Gotlib et al. 2013; Horny et al. 2008). To incorporate these data into the study, C-findings were collected, and patients were categorized as having ASM, ISM, or other SM subtype retrospectively based on WHO criteria for these SM subtypes (Horny et al. 2008). Per the WHO criteria, patients meeting SM criteria who had one or more C-findings were classified as having ASM as long as the criteria for MCL were not met.

Informed consent was obtained from all patients according to institutional guidelines. The study was conducted in accordance with the Declaration of Helsinki; the protocol and all amendments were reviewed and approved by the ethics board or institutional review board at each participating trial center.

### Dosing

Nilotinib 400 mg twice daily was selected as the study dose based on safety and tolerability and pharmacokinetic and preliminary efficacy data from the phase 1 portion of this study (H. Kantarjian et al. 2006). One treatment cycle consisted of 28 days of continuous dosing with nilotinib 400 mg twice daily. Patients remained on nilotinib treatment until disease progression or unacceptable toxicity that precluded additional therapy. Patients responding to therapy were allowed to enter an extension study after 24 months of treatment and continue receiving drug.

Dose modifications, including dose reductions and dose interruptions, were allowed for patients who developed intolerance to nilotinib. Patients with treatment interruptions longer than 21 days were discontinued from the study, except those with hematologic toxicity, in whom interruptions were permitted for up to 42 days. Patients discontinuing due to adverse events (AEs) were monitored until resolution or stabilization of the AE. All patients were monitored for AEs for 28 days after the final dose of nilotinib.

### Study objectives

The primary efficacy endpoint was the overall response rate (ORR), defined as the proportion of patients with a minor response or better lasting a minimum of 4 weeks (Valent et al. 2003). Response was evaluated using improvements in laboratory findings (for all patients) and ASM response criteria (for the ASM subgroup). For patients with ASM, response evaluations were based on findings in bone marrow, peripheral blood, and extramedullary sites of disease and improvements in C-findings (Valent et al. 2007). C-findings were defined as hematologic abnormalities [absolute neutrophil count (ANC) <1.0 × 10^9^/L, hemoglobin level <10 g/dL, or platelet count <100 × 10^9^/L], hepatomegaly with ascites and impaired liver function, portal hypertension, palpable splenomegaly with hypersplenism, malabsorption with hypoalbuminemia and/or weight loss, and skeletal lesions (such as large-sized osteolyses and/or severe osteoporosis with pathological fracture). The definitions of complete or incomplete remission and pure clinical response included complete resolution of 1 C-finding and no progression in other C-findings. Complete remission also required disappearance of organomegaly, decrease of serum tryptase level to <20 ng/mL, and loss of all mast cell infiltrates in affected organs. Incomplete remission required >50 % decrease in serum tryptase level and/or a decrease in mast cell infiltrates and/or visible reduction of organomegaly. Pure clinical response involved no response in mast cell infiltrates, serum tryptase level, or organomegaly. Good partial response was defined as >50 % regression in ≥1 C-findings and no progression in other C-findings. Minor response was defined as ≤50 % regression of ≥1 C-findings and no progression in other C-findings. Stable disease was defined by C-finding parameters falling within a constant range. Progressive disease was defined as ≥1 C-finding with progression.

Secondary efficacy endpoints included overall survival, which was estimated using the Kaplan–Meier method. The analysis of overall survival included all deaths occurring during treatment or after discontinuation of study drug. Assessment of *KIT* mutation status was performed by Sanger sequencing after polymerase chain reaction (PCR) amplification, and FIP1L1–PDGFRA translocation status was determined by nested reverse-transcriptase PCR at baseline and/or at various time points throughout the study. The ORR stratified by *KIT* D816V mutation status at baseline or at any time was assessed.

Laboratory parameters were assessed to help characterize the quality of response in responders in the ASM subgroup and in all patients with other SM subtypes. For each laboratory parameter, values were recorded at baseline and the time of best response. The “best value” was the lowest level of tryptase and mast cell burden and the highest level for other parameters. The median baseline and best value were calculated for the responder population. The difference between these median values was then used to determine the percentage improvement in each parameter.

Toxicity was assessed using the National Cancer Institute Common Terminology Criteria for Adverse Events (NCI CTCAE) version 3.0 (National Cancer Institute 2006). AEs, serious AEs (SAEs), and hematologic and nonhematologic laboratory abnormalities were assessed.

## Results

### Patient characteristics

Baseline demographic and disease characteristics of the 61 patients enrolled in the SM arm of the 2101 study are listed in Table 1. Patients were retrospectively assigned to SM subgroups based on WHO 2008 criteria (Tefferi and Vardiman 2008). The patient population was heterogeneous. Most patients had ASM [*n* = 37 (60.7 %)] or ISM [*n* = 19 (31.1 %)]; of the 5 remaining patients, 3 had MCL, 1 had SM-acute myeloid leukemia (AML), and 1 had SM with an associated clonal hematologic non–mast cell lineage disease (AHNMD). At study entry, 49 patients (80.3 %) met 1 major and 1 minor disease criteria for SM; 11 patients (18.0 %) met 3 minor criteria for SM; and 1 patient (1.6 %) had a major protocol violation, defined as entering the study but not meeting the predefined SM disease group classification. In the ASM group (*n* = 37), C-findings were as follows: 15 patients had skeletal involvement only; 11 patients had cytopenias only; 3 patients had hepatosplenomegaly only; 3 patients had skeletal lesions accompanied by hepatosplenomegaly; and 5 patients had malabsorption, skeletal lesions, and hepatosplenomegaly. The median time since first diagnosis of SM was 25.9 months (range 1.2–287.3 months). The median patient age was 52 years (range 29–79 years). The median time since diagnosis was similar in patients with ASM and those with ISM (25.9 vs 27.4 months, respectively). At baseline, 36 patients (59.0 %) had a *KIT* D816V mutation, and 5 patients (8.2 %) had no *KIT* mutation; in 20 patients (32.8 %), *KIT* mutation analysis was missing. All patients had at least 1 sample evaluated for *KIT* mutation status at any point during the study. Of the 25 patients with no or missing mutation data at baseline, 12 (48.0 %) subsequently tested positive for *KIT* D816V. Using data obtained at any point during the study, 48 patients (78.7 %) had a *KIT* D816V mutation, and 13 patients (21.3 %) did not; no patients had a D816Y mutation. All of the 53 patients tested for the FIP1L1–PDGFRα fusion at any time during the study had a negative result.

**Table 1:** Characteristics of all patients and responders

	Total	ASM		ISM	Other^a^
	All patients (*N* = 61)	All patients (*n* = 37)	Responders (*n* = 8)	All patients (*n* = 19)	All patients (*n* = 5)
Characteristics at baseline					
Median age (range, years)	52 (29–79)	49 (29–79)	55 (41–79)	50 (33–78)	66 (52–67)
Male, *n* (%)	34 (55.7)	21 (56.8)	3 (37.5)	11 (57.9)	2 (40.0)
Entry criteria for SM, *n* (%)					
Major + 1 minor criteria	49 (80.3)	29 (78.4)	8 (100)	16 (84.2)	4 (80.0)
Three minor criteria	11 (18.0)	8 (21.6)	0	2 (10.5)	1 (20)
Other^b^	1 (1.6)	0	0	1 (5.3)	0
No prior treatment, *n* (%)	29 (47.5)	15 (40.5)	1 (12.5)	10 (52.6)	4 (80.0)
WHO performance status, *n* (%)					
Grade 0	26 (42.6)	15 (40.5)	1 (12.5)	9 (47.4)	2 (40.0)
Grade 1	25 (41.0)	14 (37.8)	6 (75.0)	9 (47.7)	2 (40.0)
Grade 2	8 (13.1)	6 (16.2)	0	1 (5.3)	1 (20.0)
Grade >2	1 (1.6)	1 (2.7)	1 (12.5)	0	0
Missing	1 (1.6)	1 (2.7)	0	0	0
Median time since diagnosis of SM (range, months)	25.9 (1.2–287.3)	25.9 (1.2–287.3)	34.5 (9.2–84.8)	27.4 (1.6–123.4)	8.34 (3.1–77.6)
Time since SM diagnosis, *n* (%)					
<6 months	13 (21.3)	10 (27.0)	0	2 (10.5)	1 (20.0)
6 months to <1 year	8 (13.1)	3 (8.1)	1 (12.5)	3 (15.8)	2 (40.0)
1 to <2 years	8 (13.1)	5 (13.5)	1 (12.5)	3 (15.8)	0
2 to <5 years	13 (21.3)	9 (24.3)	3 (37.5)	3 (15.8)	1 (20.0)
≥5 years	19 (31.1)	10 (27.0)	3 (37.5)	8 (42.1)	1 (20.0)
*KIT* D816V mutation status, *n* (%)					
At baseline^c^					
Yes	36 (59.0)	22 (59.5)	6 (75.0)	10 (52.6)	4 (80.0)
No	5 (8.2)	2 (5.4)	0	3 (15.8)	0
Missing	20 (32.8)	13 (35.1)	2 (25.0)	6 (31.6)	1 (20.0)
At any time					
Yes	48 (78.7)	29 (78.4)	8 (100)	15 (78.9)	4 (80.0)
No	13 (21.3)	8 (21.6)	0	4 (21.1)	1 (20.0)
Missing	0	0	0	0	0
Laboratory values at baseline					
Tryptase level, ng/mL	(*n* = 54)	(*n* = 33)	(*n* = 7)	(*n* = 16)	(*n* = 5)
<20	4 (7.4)	3 (9.1)	1 (14.3)	1 (6.3)	0
20–100	18 (33.3)	8 (24.2)	1 (14.3)	8 (50.0)	2 (40.0)
>100–200	19 (35.2)	13 (39.4)	2 (28.6)	5 (31.3)	1 (20.0)
>200	13 (24.1)	9 (27.3)	3 (42.9)	2 (12.5)	2 (40.0)
Hemoglobin level (g/dL)					
<10	7 (11.5)	7 (18.9)	2 (25.0)	0	0
10–15	47 (77.0)	26 (70.3)	6 (75.0)	16 (84.2)	5 (100)
>15	7 (11.5)	4 (10.8)	0	3 (15.8)	0
Albumin level (g/dL)					
<3.4	7 (11.5)	5 (13.5)	2 (25.0)	0	2 (40.0)
3.4–5.4	54 (88.5)	32 (86.5)	6 (75.0)	19 (100)	3 (60.0)
>5.4	0	0	0	0	0
ANC (×10^9^/L)					
<2	4 (6.6)	3 (8.1)	1 (12.5)	0	1 (20.0)
2–7	47 (77.0)	28 (75.7)	5 (62.5)	16 (84.2)	3 (60.0)
>7	10 (16.4)	6 (16.2)	2 (25.0)	3 (15.8)	1 (20.0)
Platelet count (×10^9^/L)					
<100	9 (14.8)	8 (21.6)	2 (25.0)	0	1 (20.0)
100–400	50 (82.0)	27 (73.0)	5 (62.5)	19 (100)	4 (80.0)
>400	2 (3.3)	2 (5.4)	1 (12.5)	0	0
Bone marrow mast cell infiltration (%)	(*n* = 55)	(*n* = 34)	(*n* = 7)	(*n* = 17)	(*n* = 4)
<10	24 (43.6)	16 (47.1)	4 (57.1)	8 (47.1)	0
10–20	15 (27.3)	9 (26.5)	2 (28.6)	5 (29.4)	1 (25.0)
>20–30	5 (9.1)	2 (5.9)	1 (14.3)	3 (17.6)	0
>30	11 (20.0)	7 (20.6)	0	1 (5.9)	3 (75.0)
Symptoms at baseline, *n* (%)^d^					
Urticaria	20 (32.8)	11 (29.7)	2 (25.0)	8 (42.1)	1 (20.0)
Cutaneous symptoms	36 (59.0)	21 (56.8)	5 (62.5)	13 (68.4)	2 (40.0)
Constitutional symptoms	33 (54.1)	22 (59.5)	3 (37.5)	8 (42.1)	3 (60.0)
Mediator-related symptoms	31 (50.8)	20 (54.1)	5 (62.5)	9 (47.4)	3 (60.0)

*AML* acute myeloid leukemia, *AHNMD* associated clonal hematologic non–mast cell lineage disease, *ANC* absolute neutrophil count, *ASM* aggressive systemic mastocytosis, *ISM* indolent systemic mastocytosis, *MCL* mast cell leukemia, *SM* systemic mastocytosis, *WHO* World Health Organization

^a^Includes 3 patients with MCL, 1 patient with SM-AHNMD (responder, incomplete remission), and 1 patient with SM-AML

^b^Patient withdrew consent and was discontinued from the trial on study day 34

^c^Based on bone marrow or peripheral blood samples taken at baseline

^d^Symptoms were categorized as follows: constitutional symptoms—weight loss, fever, chills, night sweats, fatigue, lethargy, nausea, vomiting, and diarrhea; cutaneous symptoms—pruritus, flushing, urticaria, angioedema, erythema, atopic dermatitis, rashes, systemic lupus erythematosus, herpes zoster, and skin mastocytosis; mediator-related symptoms—headache, dizziness/lightheadedness, migraine, vertigo, and syncope/presyncope

Baseline characteristics of these retrospectively determined subgroups reflected differences in overall disease status. For example, the proportion of patients with WHO performance status of ≥2 was higher in the ASM group (18.9 %) than in the ISM group (5.3 %). Fewer patients in the ASM group had no prior treatment for SM (40.5 vs 52.6 % in the ISM group). Baseline tryptase levels were generally higher in the ASM group (27.3 % of patients had levels >200 ng/mL at baseline vs 12.5 % of patients in the ISM group). Anemia (hemoglobin level <10 g/dL), neutropenia (ANC <2 × 10^9^/L), and thrombocytopenia (platelet count <100 × 10^9^/L) were all more common in the ASM group than the ISM group (18.9 vs 0 %, 8.1 vs 0 %, and 21.6 vs 0 %, respectively). The proportion of patients with bone marrow mast cell infiltration >30 % was also greater in the ASM group (20.6 %) than in the ISM group (5.9 %). Constitutional and mediator-related symptoms were more common in patients with ASM than in those with ISM (59.5 vs 42.1 and 54.1 vs 47.4 %, respectively), whereas urticarial and cutaneous symptoms were less common in the ASM group than the ISM group (29.7 vs 42.1 and 56.8 vs 68.4 %, respectively).

### Patient disposition and drug exposure

At the time of data cutoff (January 19, 2009), 8 patients had entered the extension study, and 53 patients had discontinued treatment (Table 2). The most common reason for discontinuation was AEs [*n* = 18 (29.5 %)]. Diarrhea, fatigue, and pain in extremity (3.3 % each) were the AEs most frequently leading to discontinuation. Eleven patients (18.0 %) experienced grade 3/4 AEs leading to discontinuation. Each grade 3/4 AE occurred in only 1 patient. Eleven patients (18 %) had disease progression that led to discontinuation.

**Table 2:** Patient disposition

	*n* (%)
Total patients treated	61 (100)
Entered extension study^a^	8 (13.1)
Discontinued core treatment	53 (86.9)
Adverse event(s)	18 (29.5)
Withdrew consent	15 (24.6)
Disease progression	11 (18.0)
Administrative problems^b^	5 (8.2)
Abnormal test procedure result(s)	1 (1.6)
Protocol violation	1 (1.6)
Lost to follow-up	1 (1.6)
Death^c^	1 (1.6)

^a^Enrolled in the ongoing extension portion of the study at the time of data cutoff

^b^One patient underwent dose interruption for thrombocytopenia, which did not resolve within the time period specified by the protocol; the patient was therefore removed from the study by the sponsor. The other 4 patients were discontinued due to unsatisfactory response (all had stable disease)

^c^Only includes patients for whom death was cited as the primary reason for discontinuation of study treatment

The median total daily dose of nilotinib received was close to the planned total daily dose of 800 mg [787.6 mg (range 318.9–953.5 mg)]. Median treatment duration was 232 days (range 3–1274 days). One-third of patients (32.8 %) were treated with nilotinib for a minimum of 12 months, and 18.0 % received nilotinib for ≥24 months. Nearly half of all patients had a nilotinib dose reduction [*n* = 29 (47.5 %)] and/or interruption [*n* = 30 (49.2 %)]. The most common AEs requiring dose adjustment or interruption were nausea (16.4 %), diarrhea (14.8 %), vomiting (13.1 %), and fatigue (6.6 %). Dose interruptions lasted a median of 13.5 days (range 2–47 days). Interruptions constituted a median of 5.1 % of total dosing days (range 0.2–42 %).

### Efficacy and characteristics of responders in the ASM subgroup

In the ASM subgroup, a minor response or better was achieved by 8 patients (21.6 %) receiving nilotinib 400 mg twice daily, all of whom had a *KIT* D816V mutation at any time during the study (Table 3). Incomplete remission and pure clinical response were each achieved by 1 patient, while good partial response and minor response were each achieved by 3 patients (8.1 %). Stable disease was observed in 16 patients (43.2 %), and 3 patients (8.1 %) had disease progression as their best response. Ten patients were not evaluable for response, in part because some patients did not have baseline C-finding data, which were collected retrospectively after response criteria for ASM became available (Gotlib et al. 2013).

**Table 3:** Best responses in the ASM subgroup and by *KIT* D816V mutation status at baseline or at any time during the study

	ASM (*n* = 37)	*KIT* D816V mutation at baseline		*KIT* D816V mutation at any time	
		Yes (*n* = 22)	No/missing (*n* = 15)	Yes^a^ (*n* = 29)	No (*n* = 8)
Response (95 % CI), *n* (%)					
Overall response	8 (21.6)	6 (27.3)	2 (13.3)	8 (27.6)	0
Complete remission	0	0	0	0	0
Incomplete remission	1 (2.7)	0	1 (6.7)	1 (3.4)	0
Pure clinical response	1 (2.7)	1 (4.5)	0	1 (3.4)	0
Good partial response	3 (8.1)	2 (9.1)	1 (6.7)	3 (10.3)	0
Minor response	3 (8.1)	3 (13.6)	0	3 (10.3)	0
Absence of response	29 (78.4)	16 (72.7)	13 (86.7)	21 (72.4)	8 (100)
Stable disease	16 (43.2)	9 (40.9)	7 (46.7)	14 (48.3)	2 (25.0)
Progression	3 (8.1)	1 (4.5)	2 (13.3)	1 (3.4)	2 (25.0)
Not evaluable	10 (27.0)	6 (27.3)	4 (26.7)	6 (20.7)	4 (50.0)

*ASM* aggressive systemic mastocytosis

^a^Includes 7 patients with ASM with no/missing mutation data at baseline

In patients with ASM, responses were evaluated based on baseline *KIT* D816V mutation status (Table 3). Of 22 patients with mutated *KIT* D816V at baseline, 6 (27.3 %) achieved an overall response to treatment, including 1 pure clinical response (4.5 %), 2 good partial responses (9.1 %), and 3 minor responses (13.6 %). Nine patients with a baseline *KIT* D816V mutation (40.9 %) had stable disease, and 1 patient (4.5 %) had disease progression. Of 15 patients with no baseline mutation or missing *KIT* D816V status, 2 (13.3 %) had an overall response to treatment [including 1 incomplete remission (6.7 %) and 1 good partial response (6.7 %)], 46.7 % had stable disease, and 13.3 % had disease progression as their best response. However, 7 of 15 patients with no or missing *KIT* mutation status at baseline tested positive for a D816V mutation in subsequent analyses.

The baseline characteristics of ASM responders were largely similar to those of the total ASM population, but some differences were observed (Table 1). For instance, all responders had the major criterion for SM (vs 78.4 % of all patients). Further, the proportion of patients with constitutional symptoms was lower in responders than in the total ASM population (37.5 vs 59.5 %), and the proportion of patients with bone marrow mast cell infiltration <10 % was higher in responders (62.5 vs 47.1 %). The median time since SM diagnosis was longer for responders than for all patients (34.5 vs 25.9 months), and presence of the *KIT* D816V mutation at any time was more common in responders (100 %) compared with the total ASM population (78.4 %).

### Description of response as measured by changes in laboratory parameters

**Fig. 1:**
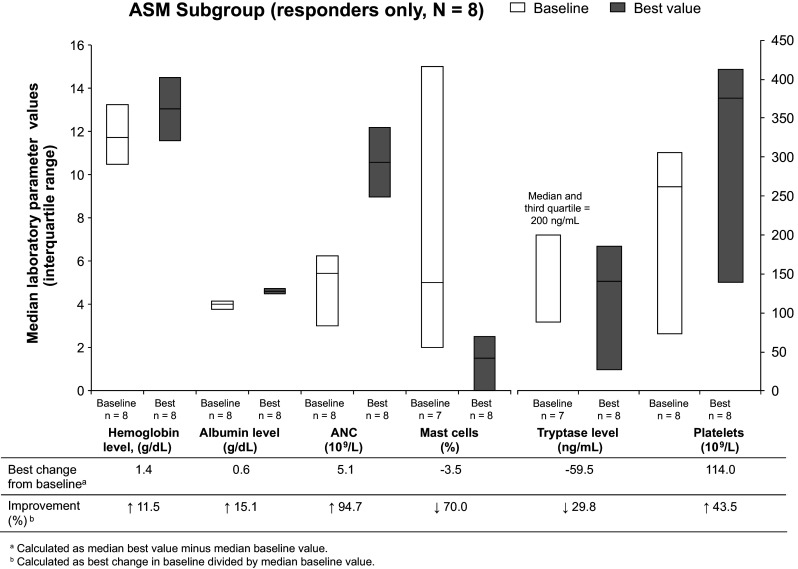
Improvements in laboratory parameters of responders in the ASM subgroup while on study

In responders in the ASM subgroup, the parameter with the highest degree of change was ANC; median best increase from baseline was 94.7 % **(**Fig. 1). Furthermore, when best values were compared with baseline values, the proportion of mast cells found in bone marrow decreased 70.0 %. Moderate change was seen in platelet counts (43.5 % increase) and tryptase levels (29.8 % decrease). There was little change in hemoglobin and albumin levels during the study (increases of 11.5 and 15.1 %, respectively).

**Fig. 2:**
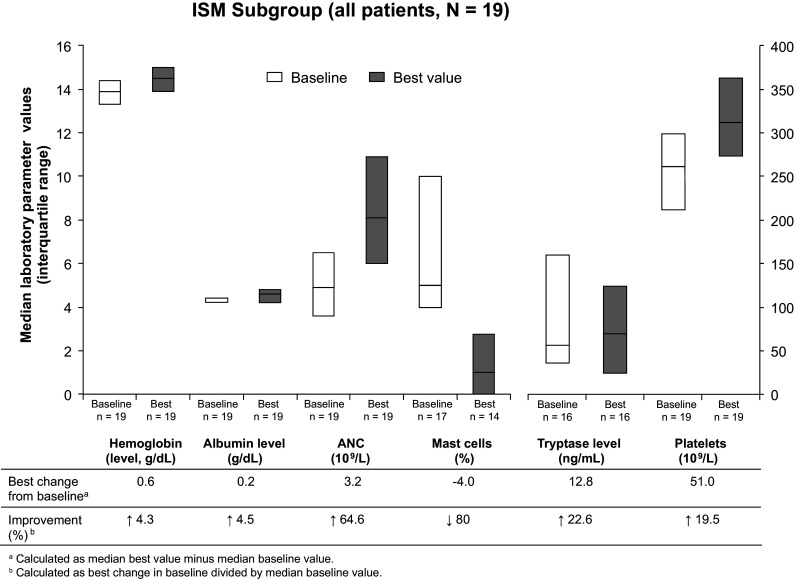
Improvements in laboratory parameters in the ISM subgroup while on study

Because the ASM criteria for response cannot be used in patients with other SM subtypes, patients in other subgroups were evaluated based on changes in laboratory parameters. In the ISM subgroup (*n* = 17 evaluable at baseline), the proportion of mast cells found in the bone marrow decreased 80 %, and ANC decreased by 64.6 % (Fig. 2). Platelet counts increased by 19.5 %. As in the ASM subgroup, hemoglobin and albumin levels showed minimal change during the study (increases of 4.3 and 4.5 %, respectively). The median tryptase level in the ISM group increased by 22.6 %; however, the interquartile range of tryptase levels at baseline and best value during the study showed a trend for decreasing tryptase levels.

**Fig. 3:**
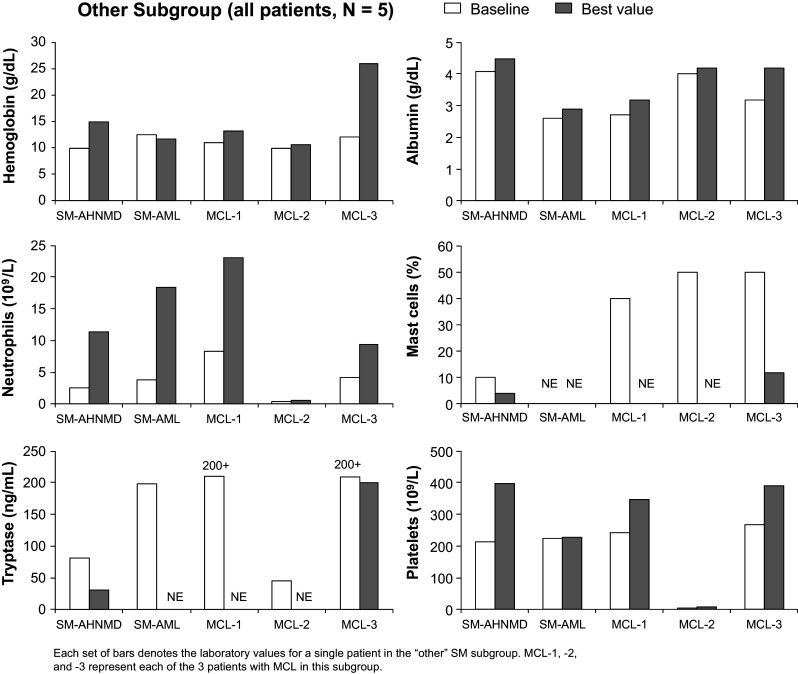
Improvements in laboratory parameters for each patient in the “other” subgroup while on study

Because patients in the “other” subgroup had different SM designations, patient laboratory values were evaluated individually rather than as a group (Fig. 3). All of these patients, except 1 patient with MCL (denoted as MCL-3 in the figure), had a *KIT* D816V mutation at any time. In general, most patients in the “other” subgroup experienced improvements in albumin and neutrophil levels and platelet counts. The patient with SM-AML experienced a decrease in hemoglobin, while all other patients experienced an increase in hemoglobin during the study. Because of missing values, trends in mast cell infiltration and tryptase level in this population could not be determined.

### Survival

**Fig. 4:**
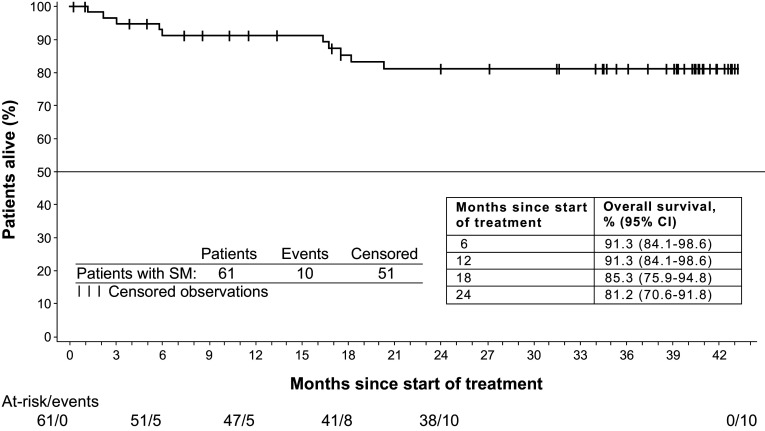
Overall survival

During the study, 11 deaths occurred: 9 in the ASM subgroup and 2 in the “other” subgroup (both patients with MCL). Four deaths occurred during treatment or within 28 days of study drug discontinuation, while 7 deaths occurred during long-term follow-up. Ten deaths were due to disease progression, and 1 cause of death was listed as dyspnea and weakness. Of patients with *KIT* D816V mutations at baseline (*n* = 36) or at any time (*n* = 48), 5 deaths were reported. The rate of overall survival at 24 months was 81.2 % (95 % CI 70.6–91.8 %), with the median overall survival not yet reached after a median of 34.7 months of follow-up (Fig. 4).

### Safety

All patients reported at least 1 AE, which was not unexpected given the severe nature of the underlying disease. The most frequent study drug-related AEs included nausea (39.3 %), headache (32.8 %), fatigue (29.5 %), diarrhea (27.9 %), vomiting (26.2 %), and pruritus (24.6 %; Table 4). Gastrointestinal and skin toxicities were consistent with the overall safety profile for nilotinib (Kantarjian et al. 2007; Tasigna package insert. 2015). Newly occurring or worsening grade 3/4 hematologic laboratory abnormalities included thrombocytopenia (10.3 %), anemia (10.0 %), and neutropenia (6.9 %; Table 5). The most frequent newly occurring or worsening grade 3/4 biochemical laboratory abnormalities included decreased serum phosphate (17.2 %), increased serum lipase (17.0 %), increased serum bilirubin (13.3 %), and increased serum alanine aminotransferase (10.2 %). Study drug-related SAEs of any grade were experienced by 14 patients (23.0 %). Diarrhea (4.9 %), vomiting (3.3 %), and dehydration (3.3 %) were the only study drug-related SAEs that occurred in more than 1 patient. Patients were carefully monitored for cardiac changes occurring on study. A QTcF increase >60 ms from baseline occurred in two patients but did not result in treatment discontinuation in either patient. No patients had a QTcF >480 ms.

**Table 4:** Most common study drug-related nonhematologic AEs (occurring in ≥10 % of patients)

Nonhematologic AE, *n* (%)	Any grade (*n* = 61)	Grade 3/4 (*n* = 61)
Nausea	24 (39.3)	2 (3.3)
Headache	20 (32.8)	3 (4.9)
Fatigue	18 (29.5)	1 (1.6)
Diarrhea	17 (27.9)	4 (6.6)
Vomiting	16 (26.2)	2 (3.3)
Pruritus	15 (24.6)	1 (1.6)
Muscle spasms	13 (21.3)	2 (3.3)
Upper abdominal pain	12 (19.7)	1 (1.6)
Rash	10 (16.4)	1 (1.6)
Dizziness	8 (13.1)	1 (1.6)
Arthralgia	7 (11.5)	1 (1.6)
Bone pain	7 (11.5)	1 (1.6)
Myalgia	7 (11.5)	1 (1.6)
Peripheral edema	7 (11.5)	1 (1.6)

*AE* adverse event

**Table 5:** Newly occurring or worsening grade 3/4 laboratory abnormalities (occurring in ≥5 % of evaluable patients)

Laboratory abnormality	*n*/*N* ^a^ (%)
Hematologic	
Thrombocytopenia	6/58 (10.3)
Anemia	6/60 (10.0)
Neutropenia	4/58 (6.9)
Nonhematologic	
Decreased serum phosphate	10/58 (17.2)
Increased serum lipase	9/53 (17.0)
Increased serum bilirubin (total)	8/60 (13.3)
Increased serum ALT	6/59 (10.2)
Decreased sodium	4/60 (6.7)
Hyperglycemia	3/56 (5.4)
Decreased serum albumin	3/58 (5.2)
Decreased potassium	3/60 (5.0)

*AE* adverse event, *ALT* alanine aminotransferase

^a^Patients evaluable postbaseline who had <grade 4 at baseline

## Discussion

SM is a heterogeneous disease. Consensus guidelines on the diagnosis and subclassification of patients with SM were published in 2007 (Valent et al. 2007) and have since been updated and refined by several groups, including the WHO (Pardanani and Tefferi 2010a, b; Valent et al. 2012; Horny et al. 2008). These criteria have established definitive categories of SM distinguishable by their clinical presentation, histology, and growth characteristics: ISM, ISM with bone marrow involvement but no skin lesions, smoldering SM (SSM), SM-AHNMD, ASM, and MCL (Gotlib et al. 2013). These categories were established after the design of the current trial. Here, disease subtypes were determined retrospectively, and the study population included patients with a heterogeneous mixture of SM subtypes, making interpretation of the data difficult.

Patients with different SM subtypes require different types of treatment. Patients with ISM may not require cytoreductive therapy because their symptom burden is low (Valent et al. 2010). Traditional treatments for more advanced SM, including cladribine (Hermine et al. 2010) and IFN (Casassus et al. 2002), are sufficient to improve disease symptoms but have limited efficacy in reducing underlying disease burden (Lim et al. 2009a). Given the inadequacy of current treatment strategies, particularly with aggressive SM subtypes, and the role that KIT activation may play in the disease, the level of interest in targeted therapies for the treatment of SM has increased.

The high prevalence of the *KIT* D816V activating mutation in patients with SM led to the development of KIT inhibitors for the treatment of this disease (Pardanani et al. 2003). Imatinib is a KIT-targeted TKI that is approved for patients with multiple hematologic malignancies, including adult patients with ASM without the *KIT* D816V mutation or with unknown *KIT* mutational status (Gleevec package insert. 2015). However, imatinib has demonstrated only modest activity against the *KIT* D816V mutation in vitro and in the clinic (Lim et al. 2009a; Vega-Ruiz et al. 2009; Gleixner et al. 2006). For example, a study including 22 patients with SM treated with imatinib demonstrated an ORR of 33.3 % in patients without the *KIT* D816V mutation, 16.7 % in patients with this mutation, and 0 % in patients with missing mutation data (Lim et al. 2009a).

Here, we report the activity of nilotinib in patients with SM enrolled in an open-label registration trial. Patients with SM, with or without the *KIT* D816V mutation, met the standard disease criteria for SM and had a clinical indication for treatment. Approximately half of the patients (47.5 %) had received no prior treatment for SM. Eight patients (21.6 %) in the ASM subgroup had a minor response or better to nilotinib. The safety profile of nilotinib in patients with SM was similar to that previously reported with nilotinib in other hematologic malignancies (Kantarjian et al. 2007; Tasigna package insert. 2015).

Although nilotinib has shown little inhibitory activity toward the *KIT* D816V/Y mutation in vitro (von Bubnoff et al. 2005; Verstovsek et al. 2006; Manley et al. 2010), the ORR in patients with ASM with the *KIT* D816V mutation at any time was higher than that in patients without the mutation at baseline [8/29 (27.6 %) vs 0/8 (0 %), respectively]. The mechanisms responsible for this are currently unknown. The D816V mutation shifts *KIT* from the inactive conformation, to which imatinib and nilotinib bind, to its active conformation (Gajiwala et al. 2009; Weisberg et al. 2005). Nilotinib-mediated inhibition of KIT signaling in nontransformed cells that support survival of malignant cells (e.g., stromal cells, mast cells, macrophages) may provide some clinical benefit, even in patients with *KIT* D816V-mutated SM (Pittoni et al. 2011). In addition, nilotinib may be active against other KIT-dependent and -independent pathways involved in the development of SM (Gleixner et al. 2011; Schwaab et al. 2013). Additional studies are needed to further explore the pathogenesis of SM and to understand the mechanisms underlying the clinical activity of nilotinib in SM. It also must be noted that 20 patients did not have *KIT* mutation results available at baseline. Thus, response rates in patients with mutated *KIT* D816V vs unmutated *KIT* must be interpreted with caution.

In the current study, all patients with ASM who responded met the major criterion for SM diagnosis (multifocal clusters of mast cells in the bone marrow) at baseline. During the study, the proportion of mast cells in the bone marrow in patients who responded decreased by 70.0 %, suggesting that nilotinib did have an effect on the underlying disease in these patients. Likewise, serum tryptase level is a minor diagnostic criterion in SM and a strong measure of disease status. Elevated serum tryptase level can predict progression to more aggressive forms of disease (Kristensen et al. 2013) and has been shown to correlate with *KIT* D816V allele burden, a marker of disease severity (Matito et al. 2013). In the current study, the median tryptase level decreased by 29.8 % in responders with ASM. Thus, while the rate of response was not high, the quality of response in patients who did respond suggests that nilotinib may have clinical benefit in some patients. Improvements in laboratory parameters were also observed in the ISM and the “other” subgroups. However, these subgroups cannot be evaluated using standard ASM criteria due to differences in the underlying disease.

The current trial had several limitations. Current response criteria, developed after the design of this study, are based on the resolution of C-findings in patients with the aggressive subtype of SM (Gotlib et al. 2013). This study included patients less likely to have C-findings at baseline; 31.1 % of patients had ISM. Using C-findings as a measure of response may not be appropriate for patients with ISM. Also, because C-findings were collected retrospectively in this study, these data may not be complete. Furthermore, the minimum response duration in this study (4 weeks) was shorter than current response duration criteria (12 weeks) (Gotlib et al. 2013), which may have increased the rates of response observed. For these reasons, the true magnitude of the clinical benefit of nilotinib in this population is difficult to determine, and it is possible that the response rates observed in this study may overestimate the true benefit of nilotinib in patients with advanced SM.

Previous studies have demonstrated a median overall survival of 24 months in patients with ASM (Lim et al. 2009b; Fuller 2012). Despite a lack of response in the majority of patients in the current study, overall survival was 81.2 % at 24 months, with the median survival not yet reached after a median of 34.7 months of patient follow-up. Thus, our data demonstrate that treatment with nilotinib may provide some clinical benefit to patients with SM. It is also possible, as our retrospective interpretation of the study population suggests, that a sufficient number of patients with ISM were included who may have skewed the overall survival results in such a manner that the true benefit is somewhat less than identified here. Additional studies will be necessary to determine whether nilotinib improves the long-term survival of patients with SM, including patients with ASM.

Several additional KIT-targeting TKIs are currently being investigated in patients with SM. Although the dual BCR-ABL/SRC TKI dasatinib inhibits the kinase activity of *KIT* D816V in vitro (Shah et al. 2006; Lombardo et al. 2004) and has demonstrated efficacy in other myeloproliferative neoplasms, such as CML (Kantarjian et al. 2010), it has displayed minimal activity in patients with SM (Verstovsek et al. 2008). Data from a phase 2 study in patients with systemic or cutaneous mastocytosis (*N* = 25) treated with the KIT/LYN kinase inhibitor masitinib, however, demonstrated an ORR of 56.0 % and modest improvements in symptoms and quality-of-life measurements after 12 weeks of treatment (Paul et al. 2010).

The multikinase inhibitor midostaurin has also demonstrated potent in vitro activity against *KIT* D816V (Gleixner et al. 2006; Weisberg et al. 2002; Fabbro et al. 2000) as well as activity in patients with advanced SM with this mutation (Gotlib et al. 2010). Phase 2 data in a cohort of 26 patients with advanced SM demonstrated an ORR of 69.2 %, and the presence of the *KIT* D816V mutation was significantly associated with achievement of a major response (Gotlib et al. 2010). Another phase 2 study used central adjudication to identify patients with ASM or MCL, thereby producing a homogenous population of patients with advanced SM according to the current definitions (Gotlib et al. 2014). In this trial, which evaluated midostaurin in patients with ASM or MCL (*n* = 89), high rates of durable responses and good tolerability were observed. The ORR was 60 % (45 % major response and 15 % partial response). The ORR was 63 % in patients with the *KIT* D816V mutation (46 of 73) and 44 % in patients without it (7 of 16). With a median follow-up of 26 months, the median duration of response was 24 months (range 11 months—not evaluable), and median OS was 29 months (range 18 months—not evaluable). Further, improvements in symptoms and quality-of-life measures were observed in all patients across all reported scales.

KIT-targeting TKIs may also be effective when used in combination with other treatments, such as chemotherapy. Treatment with the TKI dasatinib and standard chemotherapy induced hematologic remission and decreased the levels of *KIT* D816V in the mast cells of a patient with SM-AML (Ustun et al. 2009). Combined inhibition of KIT and its downstream effectors, such as PI3K or STAT5, may also be an effective strategy in SM (Harir et al. 2008; Buet et al. 2012). Several clinical trials are evaluating additional agents in patients with SM, including the interleukin 2-diphtheria toxin fusion protein denileukin diftitox and the mechanistic target of rapamycin (mTOR) inhibitor everolimus (US National Institutes of Health 2015).

Despite its limitations, this study provides additional information about this rare disease and illustrates the importance of enrolling only patients who meet strict eligibility criteria in prospective trials to ensure consistency of populations across studies. Data from the present analysis demonstrated that nilotinib has a safety profile that is consistent with that seen in previous reports and has modest clinical activity in patients with SM, particularly patients with the *KIT* D816V mutations who otherwise have limited treatment options. Nilotinib, either alone or in combination with other agents, may have a role in the treatment of these patients. However, other TKIs, which in more recent trials have shown higher rates of response in patients with advanced SM, may provide more clinical benefit for patients with more aggressive disease. Future studies of SM should use the most recent disease classification definitions to generate homogeneous patient populations and more robust data sets. Furthermore, identification of SM patient subgroups mostly likely to benefit from nilotinib may be an important approach in future studies of SM.
